# Repeated Gait Perturbation Training in Parkinson's Disease and Healthy Older Adults: A Systematic Review and Meta-Analysis

**DOI:** 10.3389/fnhum.2021.732648

**Published:** 2021-10-26

**Authors:** Femke Hulzinga, Veerle de Rond, Britt Vandendoorent, Moran Gilat, Pieter Ginis, Nicholas D'Cruz, Christian Schlenstedt, Alice Nieuwboer

**Affiliations:** ^1^Neuromotor Rehabilitation Research Group, Department of Rehabilitation Sciences, KU Leuven, Leuven, Belgium; ^2^Department of Neurology, University Hospital Schleswig-Holstein, Christian-Albrechts-University Kiel, Kiel, Germany; ^3^Institute of Interdisciplinary Exercise Science and Sports Medicine, Department Performance, Neuroscience, Therapy and Health, Medical School Hamburg, Hamburg, Germany

**Keywords:** gait adaptation, split-belt, treadmill, rehabilitation, consolidation, Parkinson's disease

## Abstract

**Background:** Gait impairments are common in healthy older adults (HOA) and people with Parkinson's disease (PwPD), especially when adaptations to the environment are required. Traditional rehabilitation programs do not typically address these adaptive gait demands in contrast to repeated gait perturbation training (RGPT). RGPT is a novel reactive form of gait training with potential for both short and long-term consolidation in HOA and PwPD. The aim of this systematic review with meta-analysis is to determine whether RGPT is more effective than non-RGPT gait training in improving gait and balance in HOA and PwPD in the short and longer term.

**Methods:** This review was conducted according to the PRISMA-guidelines and pre-registered in the PROSPERO database (CRD42020183273). Included studies tested the effects of any form of repeated perturbations during gait in HOA and PwPD on gait speed, step or stride length. Studies using balance scales or sway measures as outcomes were included in a secondary analysis. Effects of randomized controlled trials (RCT) on RGPT were pooled using a meta-analysis of final measures.

**Results:** Of the 4421 studies, eight studies were deemed eligible for review, of which six could be included in the meta-analysis, totaling 209 participants (159 PwPD and 50 HOA). The studies were all of moderate quality. The meta-analysis revealed no significant effects of RGPT over non-RGPT training on gait performance (SMD = 0.16; 95% CI = −0.18, 0.49; *Z* = 0.92; *P* = 0.36). Yet, in some individual studies, favorable effects on gait speed, step length and stride length were observed immediately after the intervention as well as after a retention period. Gait variability and asymmetry, signifying more direct outcomes of gait adaptation, also indicated favorable RGPT effects in some individual studies.

**Conclusion:** Despite some promising results, the pooled effects of RGPT on gait and balance were not significantly greater as compared to non-RGPT gait training in PwPD and HOA. However, these findings could have been driven by low statistical power. Therefore, the present review points to the imperative to conduct sufficiently powered RCT's to verify the true effects of RGPT on gait and balance in HOA and PwPD.

**Systematic Review Registration:** https://www.crd.york.ac.uk/prospero/display_record.php? Identifier: CRD42020183273.

## Introduction

Gait impairments in the aging population are related to falls and have other serious repercussions, such as that they are associated with reduced physical activity levels (Campbell et al., 1989; Seematter-Bagnoud et al., 2006). Gait impairments and their negative consequences are further exacerbated in people with Parkinson's disease (PwPD) (Bouça-Machado et al., 2020). The neuropathology of PD progressively affects the locomotor network, particularly the striatal circuitry and alters the cerebellar involvement in adapting gait to environmental changes (la Fougère et al., 2010; Bohnen and Jahn, 2013; Hinton et al., 2019). Gait adaptation is required when the gait pattern needs to be adjusted, for instance, during the transition from straight walking to a turn. Adjusting one's gait to unexpected environmental changes additionally involves reactive postural control, which is also compromised in older adults and especially in PwPD (Benatru et al., 2008; Süptitz et al., 2013). Impairments to adjust gait become more and more apparent in PwPD with disease progression. These impairments also frequently trigger episodes of freezing of gait (FOG) in PwPD (Nutt et al., 2011), thereby further increasing the risk of falling (Deandrea et al., 2010; Weaver et al., 2016). Falls are a major burden for the aging population and more so in PwPD, where approximately 60% experience repeated falls (Wood et al., 2002; Balash et al., 2005). This poses one of the most important hurdles for clinical management of PwPD, as falls and FOG are largely refractory to medication (Curtze et al., 2015; McKay et al., 2019). All of the above stresses the need for training interventions to safely improve adaptive mobility in PwPD and healthy older adults (HOA).

Several training modalities can improve ambulation and thereby decrease the risk of falling (Canning et al., 2015; Sherrington et al., 2017). Regular treadmill training has been shown to be effective in improving gait parameters, such as speed and stride length in both HOA and PwPD (Tomlinson et al., 2012; Mehrholz et al., 2016), especially when a cognitive challenge is added to the motor training (Mirelman et al., 2016). Moreover, combining balance and strength training has shown to bring benefits for mobility and falls (Sherrington et al., 2020). However, these traditional rehabilitation programs do not directly address the typical demands of ambulation in natural environments, particularly with regard to adapting to asymmetrical demands induced by the need to make turns and directional changes (Mehrholz et al., 2016).

Repeated gait perturbation training (RGPT) is a relatively novel training concept that addresses gait adaptation and reactive balance. RGPT consists of unexpected perturbations, such as push and pulls, applied by a trainer or a cable system during walking. Additionally, novel concepts of treadmill training have emerged under the impetus of technological advances. These modalities include the ability to offer translations of the walking surface (Mansfield et al., 2010), acceleration and deceleration of the treadmill or changes in gait asymmetry imposed by split-belt-treadmills, whereby the gait speed of each leg can be controlled independently (Seuthe et al., 2019). Repeated exposure to such perturbations may have lasting effects on the ability to modulate walking and reduce falls (Gerards et al., 2017). Encouragingly, our group recently evaluated a single session of split-belt training in HOA and PwPD, showing beneficial effects on gait adaptation and turning performance that were retained for at least 24 hours (D'Cruz et al., 2020; Seuthe et al., 2020).

Apart from mimicking daily life mobility, RGPT may prove beneficial by tapping into a more reactive and subconscious way of motor learning. Indeed, PwPD and HOA to a lesser degree, rely heavily on attentional strategies during gait performance as a result of reduced motor automaticity (Montero-Odasso et al., 2012; Wu et al., 2015). Consequently, they become less able to deal with consecutive attention-requiring environmental demands (Hausdorff et al., 2006). A reactive training strategy, such as RGPT, whereby participants need to adapt their gait to sudden perturbations without prior awareness of the precise timing of perturbations is thought to modulate gait automatically via cerebellar locomotor circuits, rather than overloading the cortical frontal and anterior-basal ganglia (BG) attentional reserves (Hausdorff et al., 2006; Sarter et al., 2014). In line with this notion, Marinelli et al. (2017) proposed that training, which is not relying on attentional strategies or conscious awareness of the learning process, may still be preserved in PwPD (in some paradigms) due to cerebellar compensatory contributions (Marinelli et al., 2017). As such, RGPT training may boost the compensatory cerebellar circuits, reducing attentional demand during adaptive gait in PwPD and HOA.

Following the initial reactive response to the perturbation, conscious awareness of the perturbation likely becomes involved to some degree in the control of the subsequent gait cycles. This goal-directed aspect of RGPT likely taps into the anterior BG circuits that are relatively spared in PwPD and may assist in the acquisition of new adaptive gait strategies (Marinelli et al., 2017). Unfortunately, consolidation of new motor engrams is rather impaired in PwPD due to altered processing in the posterior BG circuits (Marinelli et al., 2017), and it therefore remains to be determined how well PwPD can retain the beneficial effects of RGPT training. All in all, the training of both reactive and goal-directed processing of gait via RGPT has potential to herald larger effects on gait in PwPD than non-RGPT types of gait training. In the present review, we therefore reviewed the literature to explore the notion that RGPT may lead to improved gait and retention in PwPD and HOA by boosting “adaptive learning” pathways (Jayaram et al., 2011; Marinelli et al., 2017).

Previous reviews summarizing the effects of perturbation training focused mainly on young and older healthy adults and a combination of neurological populations (e.g., stroke, Parkinson's disease) (Mansfield et al., 2015; Gerards et al., 2017; Papadimitriou and Perry, 2017). The meta-analysis conducted by Mansfield et al. (2015), including both older adults and patients with varying neurological disorders, showed that perturbation training could significantly reduce falls when compared to control interventions without perturbation training (RR = 0.54; 95% CI = 0.34,0.85; *P* = 0.007) (Mansfield et al., 2015). These results were corroborated by Gerards et al. (2017) who concluded in their review that perturbation training is effective in reducing falls, and that treadmill-based systems and therapist-applied perturbations are likely the most feasible approaches for perturbation training (Gerards et al., 2017). The meta-analysis by Papadimitriou and Perry (2017) further showed that perturbation training reduced falls in the laboratory for both older and younger adults 6-fold compared to the non-perturbation control groups (Papadimitriou and Perry, 2017). Taken together, prior reviews indicated beneficial effects of perturbation training for reducing falls. However, no review has focused specifically on gait perturbation training in PwPD. Given that PD is a complex multi-system disorder affecting both motor and non-motor symptoms that respond variably to therapy, generalizability of findings from prior reviews on healthy adults or other neurological disorders to PD is limited. As such, in this systematic review we set out to evaluate the evidence for immediate and long-term effects of repeated gait perturbation training (i.e., RGPT) on gait outcomes in PwPD and HOA. Therefore, the aim of this systematic review with meta-analysis is to ascertain whether RGPT is more effective in improving gait performance, expressed here as improvements in gait speed, step or stride length, in HOA and PwPD, as compared to other non-perturbation-based gait interventions. In addition, we aim to assess the impact of RGPT on balance performance as a secondary endpoint. We hypothesized that, via the modulation of “adaptive learning” circuits and eventually reduced needs for attentional processing, RGPT would prove more efficacious for improving gait performance (i.e., gait speed, step length or stride length), and balance (i.e., MiniBESTest, Berg Balance Scale or postural sway) than non-RGPT gait training in both HOA and PwPD.

## Methods

### Search Strategy and Included Databases

A systematic search of the literature was conducted on 27 April 2020 in the PubMed, Embase, Medline, Web of Science and Google Scholar databases without date restriction (Bramer et al., 2017). A final screening for eligible studies was performed on 10 February 2021. The following search syntax was used (Google Scholar example): *(gait OR locomotion OR walk OR walking)* AND *(split-belt OR split belt OR splitbelt OR balance loss OR dynamic balance OR dynamic stability OR surface translation OR trip OR tripping OR slip OR slipping OR pull OR push OR perturbation OR perturbations OR perturbed OR perturb)* AND *(rehabilitation OR repeated OR repetition OR training OR program)* AND *(Parkinson's Disease OR aging OR elderly OR older adults)*. The exact search syntax used for each of the other databases is presented in the Supplementary Materials. As no consensus exists in the current literature on how to describe “perturbation” and “training,” we used a broad range of terms in our search syntax to avoid missing eligible studies (McCrum et al., 2017). The review protocol was prospectively registered in PROSPERO (CRD42020183273) and the review conducted in accordance with the Preferred Reporting Items for Systematic Reviews and Meta-analyses (PRISMA) statement (Moher et al., 2009).

## Study Selection

The inclusion criteria for the selected studies were: (1) written in the English or Dutch language; (2) intervention study (RCT and non-RCT) assessing the effect of any type of repeated, and unexpected, perturbations during walking, hereafter called RGPT; (3) presenting outcomes on HOA (mean age ≥65 years) and/or PwPD; (4) measurement of effects right after the last training session and/or retention of effects (≥24 h after the last training session); and (5) gait speed, step length or stride length obtained as either the primary or secondary outcome. Exclusion criteria were: (1) not peer-reviewed; (2) conference abstracts; (3) reviews of the literature, with or without meta-analysis or commentaries without original data; (4) perturbations not given during gait (e.g., static or optical); (5) gait only assessed during the baseline assessment without a retest after the last training session; (6) effect of training on gait speed, step length or stride length only measured during the intervention, not in separate assessment after the last training session. Since this review focuses on the effects of perturbations during gait, all other forms of perturbation training, such as perturbations in a static context or optical instead of mechanical perturbations were excluded. Studies without a randomized controlled design (RCT) were excluded from the meta-analysis. However, because of the novelty of this field, studies with repeated measures designs with or without a control group but without randomization were included in the qualitative (i.e., descriptive) analysis. This enables the evaluation of promising paradigms not yet tested in a RCT. Two reviewers independently and sequentially screened titles and abstracts (FH, BV) and full texts (FH, VR) for eligibility. Any disagreements regarding eligibility were discussed amongst the reviewers after screening, until mutual agreement was achieved and verified by a third independent reviewer (MG).

### Data Extraction and Quality Assessment

A standardized form for data extraction was used (Microsoft Excel, version 2019, Microsoft Corp. Redmond, WA) to record information about: the study population, participant demographics, details of the intervention and control conditions, study design, primary outcome measures (e.g., gait speed, step length, stride length), secondary outcome measures (e.g., balance or postural sway), and main conclusions by the study authors. In addition, information was collated to assess the studies' internal validity using the NIH National Heart, Lung, and Blood Institute's Quality Assessment Tool for Controlled Intervention Studies. This tool uses 14 criteria for assessing internal validity and potential risk of bias (National Institutes of Health, 2019). Two reviewers independently scored the internal validity (FH, VR).

### Data Synthesis and Analysis

The primary outcome measure was gait performance, expressed as the pooled outcomes of the following gait measures: gait speed, step length or stride length. When several of the outcome measures were present, only one was included in the meta-analysis based on the following predefined prioritization: (1) gait speed, (2) step length and (3) stride length. Secondary outcomes included other gait measures (i.e., asymmetry, variability), Center of Mass (CoM) measures (i.e., sway speed) and balance performance [i.e., Mini-BESTest and the Berg Balance Scale (BBS)]. If included studies had these data available in secondary analyses, these were also included in the analysis. Three secondary analyses were performed: (1) including only studies that used regular treadmill training as an active comparator to RGPT; (2) including only studies that assessed PwPD; and finally (3) including only balance outcomes. Additional data were requested from the corresponding authors if not reported in the original publication. Authors were given at least 2 weeks' time to respond to this request before the data was labeled as missing. A meta-analysis of final measures was conducted for the post and retention scores separately, in which the means and standard deviations of the scores were used to compare the pooled effects of the different interventions in the pooled population (PwPD and HOA). If only change scores were reported and the corresponding authors did not respond to the data request, the mean was determined based on the baseline mean added to the change score of the post and/or retention timepoint. This was the case for one study (Shen and Mak, 2015). Here, the standard deviation at baseline was entered as the estimate of the standard deviation at post and/or retention.

The standardized mean difference (SMD) between the intervention and control group was calculated with a random effects model using Reference Manager (RevMan, v5.4), which accounts for inter-study variance in the methods and outcome measures. Based on the SMD corresponding 95% confidence intervals, two-sided *P*-values and the main effect sizes (Z-scores) were calculated. *P*-values < 0.05 were considered statistically significant. Heterogeneity between study effects was assessed using both the χ^2^ test and *I*^2^ statistic (Higgins et al., 2003). *I*^2^ values < 25%, between 50 and 75%, and > 75% were considered as low, moderate or large heterogeneity, respectively (Higgins et al., 2003). The results were displayed in forest plots. Possible publication and selection bias were assessed using funnel plots. Where possible, effect sizes (ES) were calculated using Cohen's d.

## Results

### Study Selection

**Figure 1:**
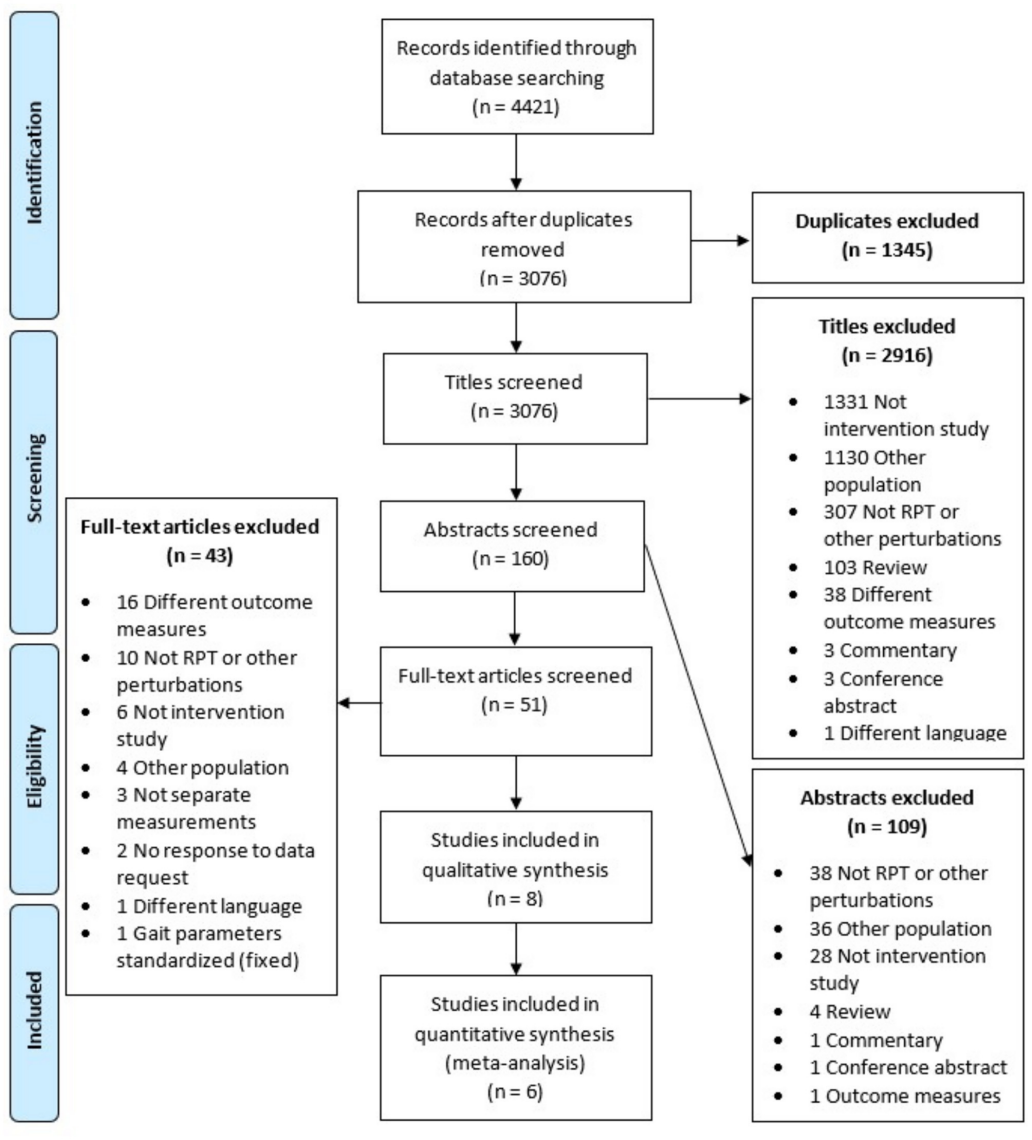
Flowchart of systematic search with the in- and exclusion and reasons per phase of the screening process. RGPT, Repeated Perturbation Training.

The search and selection procedure are outlined in Figure 1. The systematic search identified 4421 potential records. After duplicate removal, 3,076 titles were screened and 2,916 records excluded. A total of 160 abstracts were screened for eligibility, resulting in the exclusion of 109 records. Of the remaining 51 full-text records, ten met the inclusion criteria (Cakit et al., 2007; Bhatt and Yang, 2012; Yang and Pai, 2013; Harro et al., 2014a; Shen and Mak, 2015; Klamroth et al., 2016; Martelli et al., 2017; Steib et al., 2017; Gimmon et al., 2018; Rieger et al., 2020), of which two were not included as we received no response to our data requests (Bhatt and Yang, 2012; Yang and Pai, 2013). Consequently, eight studies were included in the qualitative review and six could be included in the meta-analysis for having applied an RCT design and providing use-able data (Cakit et al., 2007; Shen and Mak, 2015; Klamroth et al., 2016; Steib et al., 2017; Gimmon et al., 2018; Rieger et al., 2020). Reasons for exclusions are described in Figure 1. Two out of the eight included studies (Harro et al., 2014a; Gimmon et al., 2018) had additional data available on balance outcomes in other secondary analyses papers, which were also considered in this review (Harro et al., 2014b; Kurz et al., 2016). A summary of characteristics of the included studies can be found in Table 1.

**Table 1:** Characteristics of included studies.

**References**	**Participants age (SD)**	**Perturbation training**	**Control intervention**	**Retention**	**Gait outcome**	**Balance outcome**
Cakit et al. (2007)	*N* = 31 PwPD 71,8 (6,4) HY: 2–3 UPDRSII: 18.14 (9.32)	16 sessions, 30 min for 8 wks speed dependent treadmill training with unexpected speed increments or decrements (0.6 km/h)	Control group mentioned but content not specified	None	Max gait speed on treadmill	Berg Balance Scale
Gimmon et al. (2018)	*N* = 53 HOA IG: 78.2 (5.6) CG: 81.4 (4.3)	24 sessions, 20 min for 3 mo of treadmill at comfortable speed with unexpected perturbations of platform in random directions	24 sessions, 20 min for 3 mo of treadmill walking at comfortable speed	None	Stride length on treadmill (1.9 mph speed)	Postural sway (EC)
Klamroth et al. (2016)	*N* = 39 PwPD IG: 64.8 (10.3) HY: 2.4 (0.6) UPDRSIII: 16.7 (5.5) CG: 64.2 (8.5) HY: 2.2 (0.9) UPDRSIII: 17.7 (8.7)	1 session, 20 min of treadmill walking with small 3D tilting movements of the walking surface	1 session, 20 min of regular treadmill walking at comfortable speed	10 min	Comfortable overground gait speed, *CoV and asymm of stride length*	Postural sway (EO)
Rieger et al. (2020)	*N* = 30 HOA 72.6 (5.4)	1 session, 16 perturbations of treadmill walking with sudden acceleration or decelerations	1 session, 8 min of conventional treadmill walking	1 week	Step length on treadmill	None
Shen and Mak (2015)	*N* = 45 PwPD IG: 63.3 (8.0) HY: 2.43 (0.47) UPDRSIII: 24.0 (8.3) CG: 65.3 (8.5) HY: 2.48 (0.49) UPDRSIII: 23.2 (6.5)	44 sessions, 20–60 min, 12 wks total, 2 × 4wks 60 min of lab technology-assisted gait and balance training with volitional stepping, leaning, unexpected treadmill deceleration or manual perturbations and 1wk 20min self-supervised training.	44 sessions, 20–60 min of strength training of the lower extremity with dynamometers/leg press machines, rowing, cuff weight	3 mo (6 mo/12 mo)^*^	Comfortable overground gait speed	None
Steib et al. (2017)	*N* = 38 PwPD IG: 67.6 (8.2) HY: 2.43 (0.47) UDRSIII: 24.0 (8.3) CG: 62.5 (7.9) HY: 2.48 (0.49) UPDRSIII: 23.2 (6.5)	16 sessions, 30min for 8 wks of treadmill walking with 3D movements of tilting platform	16 sessions, 30 min for 8 wks of conventional treadmill walking	3 months	Comfortable and fast overground gait speed	Mini-BESTest, Postural sway (EO and EC)
**Paradigms without RCT design or control group**						
Harro et al. (2014a,b)^**^	*N* = 20 PwPD IG: 67.3 (11.47) HY: 1.9 (0.57) UPDRSIII: / CG: 64 (9.58) HY: 2.0 (0.67) UPDRSIII: /	18 sessions, 30 min for 6 wks of rhythmic auditory cued over-ground walking, walking to the beat of music with incremental BPM to increase gait speed	18 sessions, 30 min for 6 wks of speed dependent treadmill training, unexpected increase of speed every +/- 2.5-5 min and decreases to comfortable speed	3 months	Comfortable and fast overground gait speed	Berg Balance Scale
Martelli et al. (2017)	*N* = 18, 9 PwPD + 9 HOA PwPD: 64,3 (7,4) HY: 1.78 (0.44) UPDRSIII: 14.44 (6.44) HOA: 64,7 (7,3)	1 session, 30 min, 9 blocks of 8 AP or ML pull or push perturbations by external cables during walking on a treadmill	None	None	Step length of walking on treadmill	None

*RCT, Randomized Controlled Trial; CC, Case Control Study; IG, Intervention Group; CG, Control Group; PwPD, People with Parkinson's Disease; HOA, Healthy Older Adults; HY, Hoehn and Yahr stage; UPDRSIII, Movement Disorder Society Unified Disease Rating Scale part III; Mo, months; Wks, weeks; CoM, Center of Mass; EO, Eyes Open; EC, Eyes Closed; BPM, Beats Per Minute; AP, Anterior-Posterior; ML, Medio-Lateral; CoV, Coefficient of Variance; Asymm, asymmetry*.

* *These timepoints were also collected but not used in this meta-analysis*,

** *only control group was suitable for inclusion in qualitative analysis*.

### Summary of Study Characteristics

A total of eight studies were considered eligible for qualitative review (Cakit et al., 2007; Harro et al., 2014a; Shen and Mak, 2015; Klamroth et al., 2016; Martelli et al., 2017; Steib et al., 2017; Gimmon et al., 2018; Rieger et al., 2020), of which six could be included in the meta-analysis (Cakit et al., 2007; Shen and Mak, 2015; Klamroth et al., 2016; Steib et al., 2017; Gimmon et al., 2018; Rieger et al., 2020). Information about participants, modes of RGPT, training design, control groups, retention periods and main gait outcomes are presented in Table 1. Table 1 also illustrates the different forms of perturbations delivered while walking, including 3D tilting (Klamroth et al., 2016; Steib et al., 2017) or sudden translations of the treadmill (Steib et al., 2017; Gimmon et al., 2018), sudden acceleration or deceleration of the treadmill (Cakit et al., 2007; Harro et al., 2014a; Shen and Mak, 2015; Rieger et al., 2020), manual perturbations by the trainer (Shen and Mak, 2015), and push and pulls from a cable system during treadmill walking (Martelli et al., 2017). Six out of the eight studies compared RGPT with an active control intervention in an RCT design (Harro et al., 2014a; Shen and Mak, 2015; Klamroth et al., 2016; Steib et al., 2017; Gimmon et al., 2018; Rieger et al., 2020). Five studies used regular treadmill training (Harro et al., 2014a; Klamroth et al., 2016; Steib et al., 2017; Gimmon et al., 2018; Rieger et al., 2020) and one used strength training (Shen and Mak, 2015) as comparison. The study of Cakit et al. (2007) did not specify their control intervention.

Duration of the training sessions differed between studies. Three studies consisted of a single session (Klamroth et al., 2016; Martelli et al., 2017; Rieger et al., 2020), two studies had 16 sessions (Cakit et al., 2007; Steib et al., 2017), and the other studies provided 18 (Harro et al., 2014a), 24 (Gimmon et al., 2018) and 44 sessions, respectively (Shen and Mak, 2015), with an average of 2-3 sessions per week. The total length of the intervention period ranged from 1 day to 4 months. Retention of training effects was acquired in three studies. These studies measured retention after 1 week (Rieger et al., 2020) and 3 months (Harro et al., 2014a; Steib et al., 2017). The study of Shen and Mak (2015) measured retention effects at 3 different time points: 3, 6 and 12 months after training. To reduce heterogeneity, we included the data obtained at the 3-month time point into the retention, as this matches the retention period in two of the three studies. Five studies included PwPD (Cakit et al., 2007; Harro et al., 2014a; Shen and Mak, 2015; Klamroth et al., 2016; Steib et al., 2017), two studies included HOA (Gimmon et al., 2018; Rieger et al., 2020), and one study compared PwPD and HOA (Martelli et al., 2017). Data of both groups were pooled in the current primary meta-analysis including a total of 209 participants (159 PwPD and 50 HOA). The mean ages of PwPD and HOA differed significantly across studies [PwPD 66.4 (3.6) and HOA 76.2 (5.1), *t* = −2.807, *P* = 0.048]. The gait parameters most frequently used as an outcome were comfortable gait speed in four studies (Harro et al., 2014a; Shen and Mak, 2015; Klamroth et al., 2016; Steib et al., 2017), followed by fast gait speed in three studies (Cakit et al., 2007; Harro et al., 2014a; Steib et al., 2017), step length in two studies (Martelli et al., 2017; Rieger et al., 2020), and stride length in one study (Gimmon et al., 2018). The study of Klamroth et al. (2016) also included measures of gait variability and asymmetry (see Table 1).

### Results of the Individual Studies - Qualitative Review

Cakit et al. (2007) found a significant improvement of 0.20 m/s in maximum tolerated walking speed on the treadmill in PwPD compared to the control group (unspecified), immediately after a training with sudden accelerations and decelerations (ES = 2.15, *p* < 0.01). In addition, an improvement in balance, measured with the Dynamic Balance Scale (ES = 6.21, *p* < 0.01) and the Berg Balance scale (ES = 9.32, *p* < 0.01), and a reduction in fear of falling (*p* < 0.01) were observed (Cakit et al., 2007). Shen and Mak (2015) did not find an improvement in over-ground gait speed, but did find a significant increase in stride length in PwPD immediately (ES = 0.968, *p* = 0.003), 6 months (ES = 0.643, *p* = 0.038) and 12 months (ES = 0.783, *p* = 0.013) after their technology-assisted balance and gait training including sudden decelerations during treadmill walking (Shen and Mak, 2015). Moreover, they reported a significantly lower number of fallers after RGPT compared to the control intervention, which was retained for 12 months (*p* = 0.047) (Shen and Mak, 2015). Klamroth et al. (2016) compared 3D tilting perturbations of the treadmill platform during treadmill walking to regular treadmill walking and found a group (RGPT vs. control) by time (pre, post, retention) effect for over-ground walking speed in PwPD (ES = 0.41, *p* = 0.014). In addition, they reported a decrease in gait variability in the intervention group (ES = −0.34, *p* = 0.048), suggesting a more stable gait pattern (Klamroth et al., 2016). The studies of Steib et al. (2017), Gimmon et al. (2018), and Rieger et al. (2020) found no improvement in gait (i.e., gait speed, stride length and step length respectively) after RGPT compared to regular treadmill walking.

Two studies were not suitable for inclusion in the meta-analyses. One did not have a RCT design (Martelli et al., 2017), and the other compared two interventions of which only the control intervention matched our RGPT criteria. Here, the intervention group was focused on cueing and therefore not suitable as comparison in the meta-analysis (Harro et al., 2014a). Regardless, both studies showed a positive effect of RGPT on gait outcomes when comparing pre-post results (see Table 2). Harro et al. (2014a) found an improvement in fast gait speed directly after a training with sudden accelerations and decelerations, compared to the pre-measurement (ES = 0.46, *p* = 0.01), which was retained after 3 months (ES = 0.36, *p* = 0.05). Martelli et al. (2017) found an effect over time in step length after training participants with a push/pull cable system (ES = 0.17, *p* = 0.003), but no difference in effects between PwPD and HOA groups was demonstrated (ES = 0.33, *p* = 0.497) (Martelli et al., 2017). Retention of these effects was not measured in this study.

**Table 2:** Qualitative description of studies not included in meta-analysis.

**References**	**Main finding on gait outcome pre-post**	**Main finding on gait outcome pre-ret**
Harro et al. (2014a)	Comfortable gait speed (m/s) did improve with 4.53% after training 1.30 (0.19) vs. 1.36 (0.21), however non-significantly (*p* = 0.13). Fast gait speed (m/s) did significantly improve by 7.45% after training 1.69 (0.27) vs. 1.82 (0.30), *p* = 0.01.	Comfortable gait speed (m/s) remained increased at retention, 1.39 (0.24) vs. 1.30 (0.19), however these improvements were non-significant (*p* = 0.12). Improvements in fast gait speed retained after 3 months, 1.69 (0.27) vs. 1.80 (0.33), *p* = 0.05.
Martelli et al. (2017)	Step length (mm) increased over time in the pooled groups [HOA 14.59 (23.70), PwPD 21.78 (17.09)] (*p* = 0.003) after 30 min of perturbation training, but no group x time effect was observed (*p* = 0.497)	Not measured

*HOA, Healthy Older Adults; PwPD, People with Parkinson's Disease*.

### Effects of RGPT on Gait

#### Immediate Effects – Meta-Analysis

**Figure 2:**
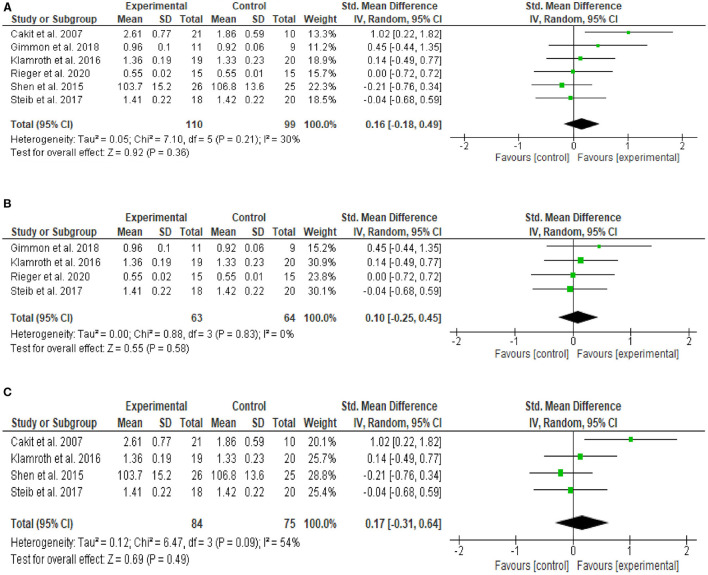
**(A)** Meta-analysis of the effect of RGPT (experimental) vs. non-RGPT (control) training on gait outcomes (i.e., gait speed, step length, or stride length) in PwPW and HOA immediately after the intervention. **(B)** Meta-analysis of the effect of RGPT (experimental) vs. regular treadmill training (control) on gait outcomes (i.e., gait speed, step length, or stride length) in PwPD and HOA immediately after training. **(C)** Meta-analysis of the effect of RGPT (experimental) vs. non-RGPT (control) training on gait outcomes (i.e., gait speed, step length, or stride length) immediately after training in PwPD only.

Figure 2A shows the outcomes of the meta-analysis of the standardized mean difference (SMD), from six studies demonstrating no improvement of gait after RGPT training compared to the control training (SMD = 0.16; 95% CI = −0.18, 0.49; *Z* = 0.92; *P* = 0.36). When pooling the data of the four studies that compared RGPT with regular treadmill training (Figure 2B), the SMD and significance level did not change considerably (SMD = 0.10; 95% CI = −0.25, 0.45; *Z* = 0.55; *P* = 0.58), although heterogeneity measures decreased (from *I*^2^ = 30% to *I*^2^ = 0%). When pooling the four studies including only PwPD, the effect size and SMD remained the same (SMD = 0.17; 95% CI = −0.31, 0.64; *Z* = 0.69; *P* = 0.49) (Figure 2C). Heterogeneity increased from 30 to 54%, reducing the robustness of these results.

#### Retention Effects – Meta-Analysis

**Figure 3:**
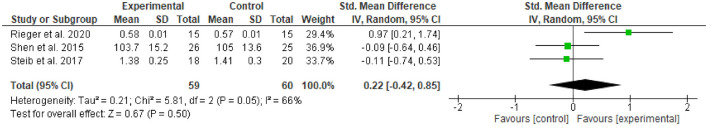
Meta-analysis of the effects of RGPT (experimental) vs. non-RGPT (control) training on gait outcomes (i.e., gait speed, step length, or stride length) in PwPD and HOA at retention.

Three studies reported the retention effects of RGPT on gait. Overall, no gait improvements were retained (SMD = 0.22; 95% CI = −0.42, 0.85; *Z* = 0.67; *P* = 0.50) (Figure 3).

### Effects of RGPT on Balance and Postural Sway

#### Immediate Effects on Balance Scales

Two studies reported the effect of RGPT on balance assessments. Cakit et al. (2007) assessed the Berg Balance Scale and Steib et al. (2017) the Mini-BESTest. Overall, RGPT showed a non-significant effect on balance assessments immediately after the training (SMD = 0.09; 95% CI = −0.40, 0.58; *Z* = 0.36; *P* = 0.72, see Supplementary Figures). These results are in line with the study of Harro et al. (2014b) that could not be included in this meta-analysis, while also showing no significant improvement on the Berg Balance Scale after RGPT at the individual study level.

#### Immediate Effects on Postural Sway

Three studies assessed velocity of postural sway during quiet stance (Klamroth et al., 2016; Steib et al., 2017; Gimmon et al., 2018). Sway was assessed with eyes open and eyes closed in one study (Steib et al., 2017) and two either assessed with eyes open (Klamroth et al., 2016) or closed (Gimmon et al., 2018). A decrease in postural sway velocity points toward better postural control. RGPT significantly decreased postural sway with eyes open (MD = −1.74; 95% CI = −3.18, −0.29; *Z* = 2.35; *P* = 0.02). Sway with eyes closed did not reduce in both studies resulting in a non-significant effect of RGPT (SMD = −1.43; 95% CI = −5.33, 2.48; *Z* = 0.72; *P* = 0.47, see Supplementary Figures).

#### Retention Effects on Balance Scales and Postural Sway

Only Steib et al. (2017) assessed 3-month retention of balance scales and postural sway. They found a slight, but statistically non-significant, decrease in Mini-BESTest scores after both the RGPT interventions in PwPD [mean difference (post-pre) = −0.1] and non-RGPT control [mean difference (post-pre) = −0.9], with lower scores indicating better performance (range from 0 to 28). The decrease in scores appears larger for the control group over the RGPT group, but no significant group by time interaction effect was found (*P* = 0.441), nor a main effect of time (*P* = 0.340). In addition, no significant improvements in postural sway with either eyes open (within group *P* = 0.862, between group *P* = 0.626), or eyes closed (within group *P* = 0.446, between group *P* = 0.626) were observed after RGPT.

### Risk of Bias in Included Studies

#### Selection Bias

Funnel plots were generated for the primary outcomes of this review immediately after the intervention and at retention and are presented in the Supplementary Materials. Both plots showed balanced heterogeneous results, pointing toward a low risk of selection bias. However, the low number of studies included may have clouded interpretation of the funnel plots.

#### Within-Study Bias

Table 3 presents the internal validity of the included studies. All studies had some risk of bias. In particular, blinding of treatment allocation (Q4) was insufficient or not reported in all studies. Four of the eight studies did blind the assessors (Q5). Nearly half of the studies reached 20% or more dropout rates (Q7), though it should be noted that these rates were often similar between intervention arms (Q8). Most importantly, only few studies justified the sample size using an a-priori power calculation (Q12). Several studies preregistered their protocols (Q13), but for most studies this was not reported. All studies assessed their outcomes using valid and reliable measures (Q11).

**Table 3:** Quality assessment of included studies.

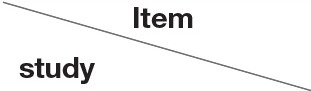	**Q1**	**Q2**	**Q3**	**Q4**	**Q5**	**Q6**	**Q7**	**Q8**	**Q9**	**Q10**	**Q11**	**Q12**	**Q13**	**Q14**
Klamroth et al. (2016)	1	1	1	0	0	1	1	1	1	?	1	0	1	1
Shen and Mak (2015)	1	1	1	0	1	1	0	1	1	?	1	1	?	0
Cakit et al. (2007)	1	?	?	?	1	1	0	0	1	?	1	0	?	0
Gimmon et al. (2018)	1	1	?	?	?	0	0	1	?	?	1	1	1	?
Steib et al. (2017)	1	1	1	0	1	1	0	1	1	1	1	0	1	0
Rieger et al. (2020)	1	?	?	?	?	1	1	1	?	?	1	0	?	1
Harro et al. (2014a)	1	?	?	0	1	1	1	1	1	?	1	1	?	1
Martelli et al. (2017)				?	?	1			1	?	1	0	?	1
**Sum score**	7/7	4/7	3/7	0/8	4/8	7/8	3/7	6/7	6/8	1/8	8/8	3/8	3/8	4/8

*Green color: low risk of bias, Red color: high risk of bias, Orange color: not reported, Dark gray color: not applicable. Q1: RCT, no/RCT, Q2: randomization quality, Q3: concealed allocation, Q4: participant blinding, Q5: blinded testers, Q6: similar groups, Q7: drop-out <20%, Q8: differential drop-out rate <15%, Q9: adherence >75%, Q10: similar background treatments, Q11: valid outcome measures, Q12: power >80%, Q13: preregistration, Q14: intention-to-treat*.

## Discussion

The aim of the present systematic review was to investigate the short and long-term effects of repeated gait perturbation training (RGPT) vs. non-RGPT training on gait performance in healthy older adults and people with Parkinson's disease. Overall, no significant additional beneficial effects of RGPT on gait performance were found when contrasted to regular treadmill training, especially for retention. Some individual studies did show favorable pre-post and between-group effects for gait speed, step length, stride length, gait variability and asymmetry measures, immediately after RGPT and after a retention period (Cakit et al., 2007; Harro et al., 2014a; Shen and Mak, 2015; Klamroth et al., 2016; Martelli et al., 2017).

Our findings are in contrast to our hypothesis and diverge from previous reviews showing a beneficial effect of repeated balance and/or gait perturbations in HOA and PwPD on reducing fall risk and increasing reactive recovery (Mansfield et al., 2015; McCrum et al., 2017), though these prior reviews did not assess gait performance. We propose three complementary explanations for the lack of significant results in the present review.

First, most of the included studies in this review contrasted RGPT with regular treadmill training (Klamroth et al., 2016; Steib et al., 2017; Gimmon et al., 2018; Rieger et al., 2020). Treadmill training alone has shown strong positive effects on gait speed and stride length in several populations, including frail older adults and PwPD (Van Ooijen et al., 2013; Mehrholz et al., 2016; Ni et al., 2018; Pereira et al., 2020). The Cochrane review conducted by Mehrholz et al. (2016) showed substantial effects of treadmill training in PwPD when compared to other interventions (e.g., stretching, dancing, resistance training, conventional therapy) on both gait speed [Mean difference (post-pre) = 0.09 m/s; 95% CI = 0.03, 0.14; *p* = 0.001] and stride length [Mean difference (post-pre) = 0.05 m; 95% CI = 0.01, 0.09; *p* = 0.01] without increased drop-out rates or adverse events. Ni et al. (2018) found similar results on exercise interventions including treadmill for PwPD. Given the positive effects of regular treadmill training, it would require large sample sizes to detect a modest effect on gait outcomes in response to RGPT when contrasted to regular treadmill training. This is supported by the results of the individual studies included in this review, showing improvements on gait, however often less than the regular treadmill control group. As a result, most included studies were probably underpowered to detect between-group differences on gait outcomes. Moreover, only 3 out of 8 included studies determined their sample sizes based on a-priori power calculation. The outcomes of the present review might help researchers to perform a power-based sample size calculation for future intervention trials on RGPT.

Second, the effectiveness of RGPT, as for most training-based interventions, is likely dependent on the dosage and task-specificity of the training. Since the dosage of the training paradigms included in this review varied greatly, this may have influenced the results and clouded the potential of some specific perturbation paradigms. Work from Karamanidis et al. (2020) showed that RGPT could improve balance recovery responses in HOA with and without neuropathology, as long as the amount of perturbations reached a certain critical threshold. This threshold theory implies that even in neurological populations such as PwPD, retention and transfer can be achieved as long as there is sufficient training exposure to reach the optimal dose-response relationship. Future studies should conduct a meta-regression analysis to delineate the impact of intervention dosage on RGPT effectiveness, once the body of work in this domain has grown. With regard to task-specificity of RGPT, the type of perturbations may have contributed to the effectiveness of RGPT on some gait measures. Optimally, the mode of perturbation should resemble complex gait as performed in daily life, such as turns and other maneuvers. Moreover, translation should be tested in over-ground as well as experimental conditions. Seuthe et al. (2020) compared several split-belt to regular treadmill walking speeds (i.e., contrasts) in HOA and PwPD gait to see which split-belt contrasts elicited the largest improvements in step length asymmetry, which were relevant for turning. They found that changing the speed ratios between the belts during one session repeatedly led to a quicker adaptation back to symmetry in step length, compared to static ratios (i.e., a constant speed reduction of one treadmill belt with either 25% or 50%). D'Cruz et al. (2020) also found that specific split-belt perturbations, especially the “changing ratios” and steady reduction by 50%, led to improved dual-task gait speed during over-ground walking and turning in place, when compared to regular treadmill training in PwPD and HOA. In addition, in both the studies of D'Cruz et al. (2020) and Seuthe et al. (2020) these improvements in turning and asymmetry were retained for 24 h. These results suggest that split-belt training with changing ratio's, when offered at optimal dosage and form, could lead to retention and transfer effects to daily gait challenges. Furthermore, the speed or contrast at which the perturbation is introduced, appears to play a role in how people learn to cope with the perturbation, and how well-they can retain learning effects (D'Cruz et al., 2020; Seuthe et al., 2020).

Third, valid outcomes that are responsive to gait adaptation, need to be adopted to capture the potential of RGPT on gait function. Gait speed, step and stride length are gait outcome measures that also improve with regular treadmill walking. One study included in this review reported additional outcomes, that could be more indicative of gait adaptation and flexibility. Klamroth et al. (2016) reported on the coefficient of variation and asymmetry of several gait parameters, including stride length and step time. Significant reductions in stride length variability and significant increases of step time symmetry were observed compared to the control group, who received regular treadmill training (Klamroth et al., 2016). These results are corroborated by another study not included in this review from Seuthe et al. (2020), who reported a significant reduction in asymmetry following split-belt perturbations after RGPT compared to regular treadmill training, whereas no improvements in gait speed were observed. These results suggest that to quantify gait adaptation, future studies should consider incorporating gait adaptation tasks and testing the validity of asymmetry and other variability/adaptation measures of gait as outcomes of interest. In addition, validity studies are needed to test whether outcomes, which capture change immediately after imposing perturbations and after retention, are correlated with ecological gait measures.

Finally, previous results of RGPT on balance are also in sharp contradiction to our secondary analysis on balance outcomes, in which we overall found non-significant results for both sway and balance scales. However, this is likely caused by the limited number of studies, as we only included papers that primarily focused on gait perturbation and outcomes. Careful interpretation of the secondary analyses is also warranted, given that these included some of the same study participants.

The present results challenged our hypothesis that exposing people to RGPT would lead to additional gait improvements that were better retained compared to non-RGPT. Gait adaptation is likely governed by cerebellum-motor cortex connectivity (Jayaram et al., 2011; Spampinato et al., 2017). When a discrepancy occurs between the expected and experienced situation (i.e., sensory prediction error), sensory integration is facilitated by the cerebellum, allowing adaptation of motor control (Krakauer et al., 2019). Because the cerebellum is intact in HOA and not severely affected in the early disease stages of PwPD (Wu and Hallett, 2013), the functional circuits related to this structure may still have some capacity to induce learning effects relevant for gait adaptation training (Gilat et al., 2019). A recent ALE meta-analysis on fMRI findings showed that PwPD consistently activate the cerebellar locomotor region more than HOA during gait, supporting the view that the cerebellum plays an important compensatory role in gait processing for PwPD (Gilat et al., 2019). In patients with cerebellar lesions, Morton and Bastian (2006) showed that an intact cerebellum is essential for adaptive gait control during split-belt walking. Moreover, a recent PET imaging study showed increased lateral cerebellar activity while adjusting gait during split-belt walking in healthy young adults (Hinton et al., 2019). These imaging studies further endorse that PwPD may still be able to train gait adaptation through RGPT, constituting promising gait rehabilitation strategies for these fall-prone patients.

### Clinical Implications

Although, the results of this review were negative, it is interesting to see that five RGPT paradigms resulted in significant within-group, and sometimes between-group improvements in gait, albeit in different outcomes. Of these five interventions, three consisted of sudden accelerations or decelerations of the treadmill, either at once or in a split-belt context, requiring an immediate response (Cakit et al., 2007; Harro et al., 2014b; Shen and Mak, 2015). The similarities in these programs suggest, that also in regular clinical practice, even without specific instrumentation, it may be useful to offer training conditions that require speed changes to improve steady gait and gait flexibility. In addition, the only study that tested long-term effects showed retention of up to 12 months following RGPT (Shen and Mak, 2015) and split-belt training (D'Cruz et al., 2020; Seuthe et al., 2020) demonstrated transfer to an over-ground adaptive task, namely turning. However, the experiments included were still at the proof-of-concept stage, as our quality assessment indicated largely underpowered samples and other potential risks of bias. Inherently, the present review could thus only include few studies and with small samples, limiting the statistical power of our meta-analyses. In addition, for the main meta-analysis, data of PwPD and HOA were pooled although there was a difference in mean age, which could have biased the results. This methodology was based on an a-priory decision (see pre-registration) to not arbitrarily restrict age for PwPD and allow for optimal power in the meta-analysis. Taken together, the present review points to the need for more well-designed, adequately powered RCTs, as well as, to gap in knowledge on the impact of RGPT on daily-life ambulation, before wide implementation in the clinical field can be recommended. Future studies should also elucidate the specific type of perturbations and dosage for use in rehabilitation, to improve flexibility of gait and balance performance in older and neurological populations.

## Conclusion

This systematic review with meta-analysis on RGPT showed that despite the promising effects reported in individual studies, their pooled effects were not helpful in improving gait outcomes when compared to other training interventions. The limited number of studies, methodological heterogeneity in the type and dosage of training and the varying outcome measures further clouded possible intervention effects. However, this review also revealed the potential of RGPT, providing a rationale for conducting future effect studies in this training concept in HOA and in PwPD.

## Data Availability Statement

The original contributions presented in the study are included in the article/Supplementary Material, further inquiries can be directed to the corresponding author/s.

## Author Contributions

FH: conception, organization, and execution of the research project, acquisition and processing of the data, design and execution of the statistical analysis, writing of the first draft of the manuscript. VR and BV: execution of the research project, acquisition and processing of the data, review and critique of the manuscript. MG: execution of the research project, processing of the data, review and critique of the statistical analysis, review and critique of the manuscript. PG: execution of the research project, review and critique of the statistical analysis, review and critique of the manuscript. ND'C and CS: execution of the research project, review and critique of the manuscript. AN: conception, organization, and execution of the research project, design and review and critique of the statistical analysis, review and critique of the manuscript. All authors contributed to the article and approved the submitted version.

## Funding

This work was funded by the Jacques & Gloria Gossweiler Foundation, Switzerland and Research Foundation - Flanders (FWO) (project 11B5421N). FH was funded by Research Foundation - Flanders (FWO, project 11B5421N). FH, ND'C, CS, and AN were funded by the Jacques and Gloria Gossweiler Foundation, Switzerland. MG and VR were funded by the European Union's Horizon 2020 research and innovation programme under the Marie Sklodowska-Curie grant agreement No. 838576 (MG) and No. 721577 (VR). PG was funded by the EU Horizon IMI, the Michael J. Fox Foundation and the Internal Fund KU Leuven.

## Conflict of Interest

The authors declare that the research was conducted in the absence of any commercial or financial relationships that could be construed as a potential conflict of interest.

## Publisher's Note

All claims expressed in this article are solely those of the authors and do not necessarily represent those of their affiliated organizations, or those of the publisher, the editors and the reviewers. Any product that may be evaluated in this article, or claim that may be made by its manufacturer, is not guaranteed or endorsed by the publisher.
