# Supplementary material for: Repeated Gait Perturbation Training in Parkinson's Disease and Healthy Older Adults: A Systematic Review and Meta-Analysis
Source: Front Hum Neurosci. 2021 Oct 26;15:732648. doi: 10.3389/fnhum.2021.732648 (PMC8576267; doi:10.3389/fnhum.2021.732648)
Supplement: Supplementary file 1 [file Data_Sheet_1.DOCX]

Supplementary Material

# Search strategies used per database

***Pubmed:*** ("gait"[MeSH Terms] OR "gait"[All Fields] OR "locomotion"[All Fields] OR "walking"[All Fields] OR "walk"[All Fields]) AND ("split-belt" [All Fields] OR "split belt" [All Fields] OR splitbelt [All Fields] OR "balance loss" [All Fields] OR "dynamic balance" [All Fields] OR "dynamic stability" [All Fields] OR "surface translation"[All Fields] OR "trip" [All Fields] OR tripping [All Fields] OR slip [All Fields] OR slipping [All Fields] OR slips (All Fields) OR pull [All Fields] OR push [All Fields] OR perturbation [All Fields] OR perturbations [All Fields] OR perturbed [All Fields] OR perturb [All Fields]) AND ("Parkinson disease" [MeSH Terms] OR Parkinson [All Fields] OR aging [All Fields] OR ageing [All Fields] OR aged [All Fields] OR elderly [All Fields] OR "older adults"[All Fields]) AND (rehabilitation [All Fields] OR repeated [All Fields] OR repetition [All Fields] OR education [All Fields] OR training [All Fields] OR program[All Fields])

***Embase:*** ('gait' OR 'gait'/exp OR gait OR 'locomotion' OR 'locomotion'/exp OR locomotion OR walk OR 'walking' OR 'walking'/exp OR walking) AND ('split-belt' OR 'split belt' OR splitbelt OR 'balance loss' OR 'dynamic balance'/exp OR 'dynamic balance' OR 'dynamic stability'/exp OR 'dynamic stability' OR 'surface translation' OR trip OR 'tripping' OR 'tripping'/exp OR tripping OR 'slip' OR 'slip'/exp OR slip OR 'slipping' OR 'slipping'/exp OR slipping OR pull OR push OR perturbation OR perturbations OR perturbed OR perturb) AND (parkinson* OR 'aging' OR 'aging'/exp OR aging OR 'ageing' OR 'ageing'/exp OR ageing OR 'elderly' OR 'elderly'/exp OR elderly OR 'older adults'/exp OR 'older adults') AND ('rehabilitation' OR 'rehabilitation'/exp OR rehabilitation OR repeated OR 'repetition' OR 'repetition'/exp OR repetition OR 'training' OR 'training'/exp OR training OR 'program' OR 'program'/exp OR program) AND ([article]/lim OR [article in press]/lim OR [conference paper]/lim OR [review]/lim) AND ([embase]/lim OR [medline]/lim)

***Web of Science:*** (ALL=((((gait OR locomotion OR walk OR walking) AND ("split-belt" OR "split belt" OR splitbelt OR "balance loss" OR "dynamic balance" OR "dynamic stability" OR "surface translation" OR trip OR tripping OR slip OR slipping OR pull OR push OR perturbation OR perturbations OR perturbed OR perturb) AND (parkinson* OR aging OR ageing OR elderly OR "older adults") AND (rehabilitation OR repeated OR repetition OR training OR program))))) AND DOCUMENT TYPES: (Article OR Review)

***Google Scholar:*** (gait OR locomotion OR walk OR walking) AND ("split-belt" OR "split belt" OR splitbelt OR "balance loss" OR "dynamic balance" OR "dynamic stability" OR "surface translation" OR trip OR tripping OR slip OR slipping OR pull OR push OR perturbation OR perturbations OR perturbed OR perturb) AND (Parkinson OR ageing OR elderly OR "older adults") AND (rehabilitation OR repeated OR repetition OR training OR program)

# Supplementary Figures

## Effect of RPT on balance and postural sway


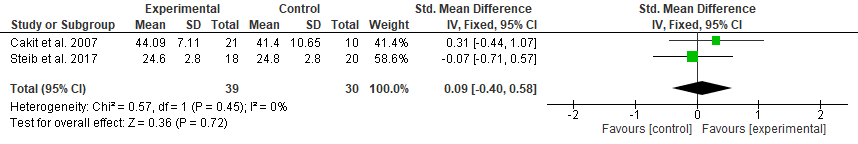


**Supplementary Figure 1.** Meta-analysis of the effect of RPT (experimental) versus non-RPT (control) training on balance scales immediately after training.


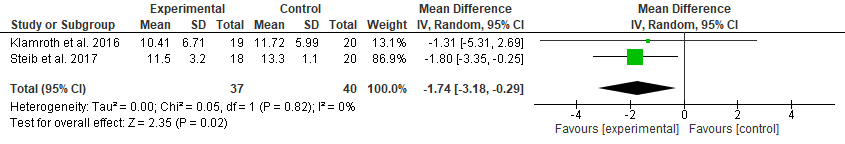


**Supplementary Figure 2.** Meta-analysis of the effect of RPT (experimental) versus non-RPT (control) training on postural sway with eyes open immediately after training.


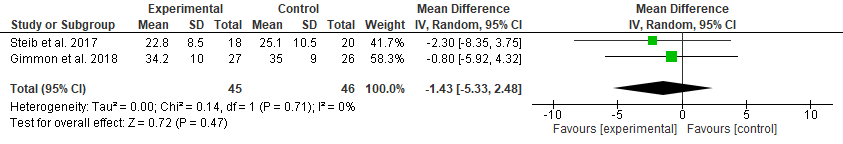


**Supplementary Figure 3.** Meta-analysis of the effect of RPT (experimental) versus non-RPT (control) training on postural sway with eyes closed immediately after training.

## Funnel plots for the primary outcome immediately after the intervention and at retention

**A**


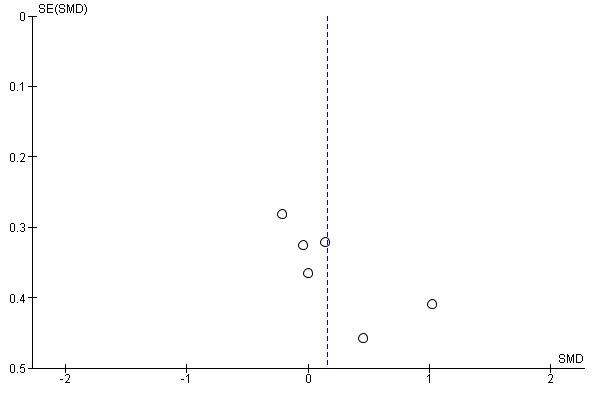


**B**


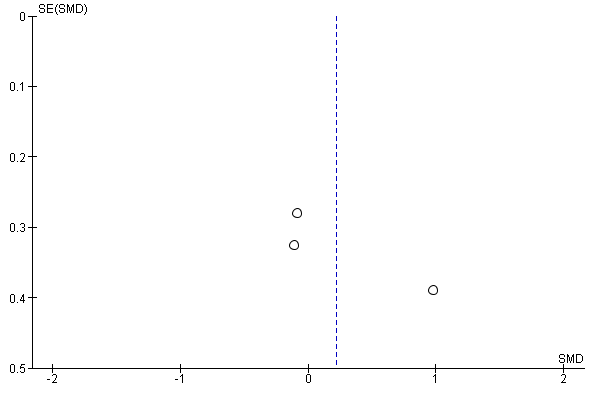
**Supplementary Figure 3.** Funnel plot primary outcome (gait performance) immediately after intervention **(A)** and after retention period **(B)**.
